# Compulsory Interventions Are Challenging the Identity of Psychiatry

**DOI:** 10.3389/fpsyt.2019.00783

**Published:** 2019-11-06

**Authors:** Paul Hoff

**Affiliations:** Department of Psychiatry, Psychotherapy and Psychosomatics, University Hospital of Psychiatry Zurich, Zurich, Switzerland

**Keywords:** autonomy, professional identity, identity of psychiatry, ethics in psychiatry, compulsory interventions

## Abstract

Compulsory interventions severely restrict constitutional rights of the patients. They are exceptional measures only to be considered under strict and clearly defined ethical and juridical conditions. They do confront mental health professionals with difficult questions challenging their individual professional identity as well as the identity of psychiatry in general. This complex field is discussed in reference to the conceptual history of psychiatry, to different contemporary approaches to the notion of autonomy, and to three ethically demanding issues: autonomy and care, psychiatry and society, personhood and interpersonal relations. Engaging open mindedly in these debates may be cumbersome for psychiatry, but will yield a substantial return, particularly regarding its identity and acceptance by society.

## Introduction

The issue of compulsory interventions in psychiatry is usually regarded as a mainly ethical and practical topic. However, this paper will link it with the fundamental question of psychiatry’s identity as a medical field in clinical and scientific contexts. In recent years—albeit, of course, not for the first time in the history of psychiatry—this conceptual area generated intense debate and controversy. Two different epistemological levels are distinctively intertwined: the *theoretical level* reflecting upon the “object” of psychiatric work in therapeutic or research activities on the one hand, and the *practical level* focusing on how psychiatric services can be optimally organized within the competing demands of being effective, adequate, and economically justifiable^1^ on the other hand. Both will now be briefly illustrated as for their conjunction with compulsory interventions.

Psychiatry’s self-understanding is challenged on the *theoretical level* when trying to define the proper “object” the field is dealing with: Is it the individual person (biographical and hermeneutic approaches), the person’s bodily existence, especially his or her central nervous system (neuroscientific approaches), or the person’s environment, ranging from close relationships to society as a whole?

On the *practical level*, the last decades brought about a remarkable paradigm shift from strong, if, of course, benevolent paternalistic attitudes to an explicit emphasis on patient autonomy and informed consent, both in clinical work and in research. Such a strong and, at times, poorly reflected notion of autonomy has been questioned repeatedly by indicating potentially negative consequences of “overvaluing autonomous decision making” (1, 2). In this context, the issue of “open psychiatry” is to be mentioned. At present, there is a broad consensus that the plain postulate to “open the wards” cannot be sufficient, unless it takes specific local conditions (including regulatory ones) into account. Furthermore, the United Nations’ “Convention on the Rights of Persons with Disabilities” (3) prominently represents the new paradigm: Not the handicapped person has to prove his or her ability to be reintegrated into society, but—the other way round—it is society’s responsibility to argue why any person, with or without handicap, should *not* be its unquestioned, in a way “normal” part^2^.

This demonstrates that any psychiatric work implicates epistemological, ethical, and anthropological (to sum up: philosophical) questions. Psychiatry has to face them, especially when tackling conflictuous topics like compulsory interventions. This, however, does not mean that philosophical considerations should be given too much weight to the disadvantage of practical issues in psychiatric services. On the contrary, if, at this point, listening to Karl Jaspers (1883–1969) who thoroughly knew both worlds, the psychiatric and the philosophical one (4), one might be surprised to come across a decisively cautious, if not critical perspective:

“The reason why the psychopathologist should care about philosophy is not that it will teach him anything positive for his own scientific field, but that it will provide him with the inner space to realize what knowledge he can possibly acquire.” (5, p.40) [translated by P.H.].

In other words, philosophical reflection itself may well be seen as necessary but not as sufficient precondition for good clinical practice in psychiatry, including the issue of responsibly handling compulsory interventions.

Before turning to three fields of tension that are practically relevant and wield major influence over the identity of psychiatry, the concept of autonomy, central to modern medical ethics, shall be highlighted (6).

## The Concept of Autonomy in Psychiatry: Essential and Cumbersome

The understanding of persons with a mental disorder as patients, as suffering individuals entitled to be taken seriously and treated efficiently, is, from a historical point of view, a comparably young concept—as is psychiatry itself: Both emerged in the era of enlightenment in the eighteenth century with its strong emphasis on rationality and personhood, that is, the notion of rationality created an optimistic stance over the scientific comprehensibility, not to say mastery of our world. The concept of personhood postulated that human individuals are not more or less passive elements of given social or political structures like kingdoms, religions, or nations, but possess a dignity of their own which includes autonomous and responsible decision making. Immanuel Kant’s (1724–1804) philosophy is the most prominent representative of such an anthropological framework. Pars pro toto, the categorical imperative, central to Kantian ethics, shall be mentioned because of its close links to the notion of autonomy:

“Act in such a way that you treat humanity, whether in your own person or in the person of any other, never merely as a means to an end, but always at the same time as an end.” (7).

To see another person *only* as a means to my own intentions is therefore disrespectful, ignores his or her autonomy, and cannot be ethically justifiable.

Thus, we may, with good reasons, trace core elements of modern psychiatry back to the evolving liberal ideas of the eighteenth century and their focus on autonomous persons and their indispensable civil rights.

However, one-sidedness must be avoided: As often in the history of science, new paradigms brought about progress *and* carried risks. It was exactly this caution that led Max Horkheimer (1895–1973) and Theodor W. Adorno (1903–1969), leaders of the “Frankfurt School,” to coin the term “dialectics of enlightenment” (8, 9): Emancipatory ideas, that are not continuously monitored and recalibrated, may be misunderstood or abused, in the worst case creating the opposite effect they had intended. Although mainly applicable to historical and political domains, this argument is also of value for psychiatry: The concept of personal autonomy, adopted from the discourse of enlightenment in the late eighteenth and early nineteenth centuries, by no means guaranteed the reduction or abolishment of compulsory measures or inhumane treatments of mentally ill people.

Another plea is raised against linking psychiatry with the liberal, person-oriented mainstream of enlightenment: Can this position ever gain credibility when psychiatry’s active support of the grossly inhumane, better, perverted, and pseudoscientific activities of medicine in the Nazi era is taken into account (10, 11)?

It has a lot to do with this dialectics of science that postmodern philosophy stayed profoundly skeptical towards “grand theories” in general and the notion of an autonomous subject as anthropological hallmark in particular. This again had instant consequences for the autonomy debate in medical ethics: In the last decades, concepts with strong, some say metaphysical presuppositions like in Kant’s deontological ethics were criticized as narrowing down normative issues to the western (Christian and liberal) tradition.

In the second half of the twentieth century and up to now, among others, two radically different alternatives were developed.

The first one defined autonomy (in the sense of free personal decision making) as the very center of what is called *conditio humana*. For existentialist philosophers, the most prominent one in this context being Jean-Paul Sartre (1905–1980), humans do not only possess the ability or the *option* to act autonomously, but also *must* decide for themselves; they are, in a way, forced to use their freedom. This, nota bene, is a formal argument. It does not address the issue of *what* the individual person choses or whether his or her choice is wise or silly, good or bad. It also is a radically individualistic approach: Any person decides, must decide for himself or herself without having to refer to a given normative framework.

The second approach denies the purely individualistic nature of autonomy, which, on the contrary, is defined as essentially interpersonal. In this view, autonomy is neither depending on metaphysical principles nor on radical individual freedom but develops and, in a way, exists only when persons communicate with each other on the basis of mutual acceptance and respect. An existentialistic author, Emmanuel Levinas (1906–1995), tried to bridge both ways of thinking about autonomy by introducing an insurmountable gap between “me” and “the other,” the latter understood as an existentially necessary element, that is radically different and not fully comprehensible to me. Therefore, Levinas’ ethics must not be mistaken as a dialogical approach like the one that, in contrast, gave distinction to Martin Buber’s (1878–1965) or Harry Stack Sullivan’s (1892–1949) work (12, 13) (see section "Personhood and Interpersonal Relations").

In recent years, “grand theory-oriented” approaches like Kantian or existentialist concepts have significantly lost influence, especially in medical ethics. Many present-day authors do accept *personal autonomy* as an essential attribute of human beings, an attribute, however, that exerts its full strength only when positioned in a communicative social context: Autonomy is not perceived as a preexisting or metaphysical idea, but originates *within* social relationships, e.g., in medical care. This basic assumption is the common denominator of broadly discussed concepts like “relational autonomy,” “ethics of care,” or “narrative ethics” (14–18).

This demonstrates the complexity and diversity of the present philosophical debate on autonomy. There seems to be, however, a minimal consensus between most of the competing approaches when it comes to medical ethics: Whatever the philosophical underpinning may be, ethically sound decision making requires respect for the other person’s opinion exactly because he or she *is a person*. Of course, respecting an opinion does not mean consenting to it. But without mutual respect, patient autonomy cannot be adequately put into practice.

Returning to the field of psychiatry, it can be stated that the notion of autonomy—the patient’s as well as the professional’s—is a core element that interconnects clinical work, psychiatric research, and the identity of the field itself (19). Here, identity does not refer to a formal philosophical context but addresses the self-understanding of people working in (and thus creating) the mental health area. Taking the conceptual history of psychiatry into account, especially the early referral to the notion of personal autonomy in the eighteenth century, compulsory interventions, i.e., overriding a person’s wishes and decisions, pose the strongest possible contrast—and lead to difficult questions for all people involved. Of course, these problems also exist in other medical areas, e.g., intensive care or pediatrics. However, in psychiatry, the continuous reflection on how psychopathological phenomena may restrict the patient’s ability to make full use of his or her autonomy is present beyond special situations like the emergency room or decisions in a palliative context: It is an indispensable element of psychiatric work in general.

Therefore, it is not just an option but a mandatory task to encourage the debate on autonomy within psychiatric institutions, laboratories, and lecture halls. In the following, this postulate will be exemplified in reference to compulsory interventions.

## Compulsory Interventions: Fields of Tension, Challenging the Identity of Psychiatry

Compulsory interventions create multiple fields of tension that impose considerable pressure on psychiatry’s identity. Three of them are to be discussed in some detail here: The ethical dilemma of autonomy versus care, the interrelation of psychiatry and society, personhood and interpersonal relations as conceptual constituents of psychiatry. Of course, these areas are substantially intertwined, but they are not synonymous. It is a demanding task for *any* psychiatric activity to address and combine them in a reasonable, person-centered manner.

### The Ethical Dilemma of Autonomy Versus Care

Two fundamental values in medicine collide in clinical situations where compulsory measures are considered: The patient’s autonomy on the one hand and his or her entitlement^3^ to an efficient treatment on the other hand. If the decision-making capacity^4^ is not reduced, which is the case in the majority of medical situations, no problem arises: The patient’s decision has to be respected, as long as it is based upon an informed consent or dissent. Given an informed *dissent*, compulsory measures, as a rule, are not allowed from a juridical and not justifiable from an ethical point of view^5^.

If, however, psychopathological phenomena severely impair the patient’s capacity to decide according to his or her intentions and preferences, the psychiatrist has to solve the arising ethical dilemma by ascribing more weight to one of the above mentioned conflicting values. Deciding about the patient’s power of judgement based on an exhaustive psychopathological examination is a complex and responsible task that psychiatrists are confronted with on a daily basis. What is more, to decide on the tenability of compulsory interventions regularly includes a prediction of possible risks that result from the existing psychopathological condition, risks for the patient or for others. Depending on the juridical context, the psychiatrist may have to seek permission by a court when considering compulsory measures but, in the first place, cannot escape the *personal* decision whether it is justifiable or not to force certain procedures upon the patient.

In the present context, dichotomous perspectives—patient and psychiatrist—are prevailing. Nonetheless, any therapist will try hard to build a therapeutic relationship even under the difficult, if not paradoxical, conditions of compulsory interventions. This directly alludes to the self-understanding of psychiatric professionals and, more general, to the identity of psychiatry. The important step from a dichotomous to an interpersonal perspective will be addressed later.

### The Interrelation of Psychiatry and Society

Compared to other medical specialties, psychiatry probably has the broadest and most complex interface with social and cultural issues. One reason is that in psychiatry the “object” of treatment and research indeed is not only an object in a quantitative-empirical sense, but the mentally ill person as a whole. Therefore, inevitably, all dimensions of personhood are involved: biological, psycho(patho)logical, social, and spiritual levels are covered by the binding mandate society awards to psychiatry. This mandate, however, is not a straightforward one. Indeed, it contains complex and even contradictory conceptual layers that may well question psychiatry’s identity.

The following examples shall further elucidate this:

– Given its particular “object” mentioned above, psychiatry has to accept that philosophical, anthropological, and also political issues will necessarily leave their marks in its own realm: cultural imprints on psychiatric nosology, mind–body problem, competing perspectives of natural sciences and humanities in research, and, last but not the least, the risk of political abuse of psychiatry, to mention a few.
– Our field has always seen controversial debates on whether or not psychiatry should accept a significant role in public policy, especially regarding decisions about compulsory hospitalization or treatment. In Switzerland, for example, the head physician of a psychiatric institution is entitled by law to order a compulsory treatment under certain conditions (involuntary admission, power of judgement lacking due to a mental disorder, no less invasive alternative available).^6^ Some argue that such a massive restriction of human rights should *without exception* be ordered by a judge, not a physician. Others doubt that a mandatory and time-consuming involvement of a juridical person will be favorable for the patient, since only the psychiatrist is skilled to quickly and substantially decide about the best option in an emergency situation. Of course, there is no simple answer to this dilemma. However, any professional person, who takes part in the diagnosis and treatment of psychiatric patients, has to seriously look into this subject.
– Society’s attitude toward psychiatry tends to be ambivalent: It commissions psychiatry to deal with people behaving in a peculiar way or reporting distressing subjective experiences like a severely depressive affect, delusional ideas, or hallucinations. The same society, however, often displays a skeptical, if not distrustful, stance on psychiatry, being influenced by negative (and stigmatizing) stereotypes about mental disorders. This tension necessarily affects people who work in psychiatry—and their professional identity.

### Personhood and Interpersonal Relations as Conceptual Constituents of Psychiatry

At present, as shown above, the ethical debate on autonomy places considerable emphasis on the interpersonal domain, e.g., in care ethics or narrative ethics. This is remarkably parallel to the conceptual history of psychiatric thinking: Initially drawing on postulates of eighteenth century enlightenment, psychiatry ever since debated the role of personal autonomy and its conjunction with the interpersonal realm. For Immanuel Kant, the subject’s personal freedom necessarily depended on the acceptance of other subjects as equally autonomous^7^. His philosophical system, being highly abstract and confined to transcendental idealism, as it was, did not exert sustained influence on psychiatry (21). However, interpersonal processes as relevant factors in each psychiatric therapy did become an acknowledged object of debate and research up to the present time.

For Karl Jaspers, psychiatric diagnosis and therapy were not just the application of certain techniques, but distinctively imbedded into an interpersonal relationship (5). The American psychiatrist Harry Stuck Sullivan (1892–1949) placed this idea in the very center of his “Interpersonal Theory of Psychiatry” (published posthumously in 1953) (13). The Austrian Jewish philosopher Martin Buber (1878–1965) and the American psychologist Carl Rogers (1902–1987) who developed “client-centered therapy” (22) met in 1957 for a—later famous—dialogue about interpersonal relations (23). Recently, the expanding field of social neuroscience became influential for psychiatry by combining neuroscientific laboratory methods with empirical data about the social (and this also means: the interpersonal) dimension of probands or patients (24).

Modern approaches in clinical medicine to strengthen patient autonomy are manifold, e.g., shared decision making (25), empowerment (26), assisted autonomy (27) (instead of substituted autonomy)^8^, advance care planning (28, 29), advance directives (30), and ethical guidelines for compulsory interventions (31, 32). It is no coincidence that most of them reach well beyond the plain dichotomy between patient and psychiatrist discussed earlier, but emphasize the vigor of the interpersonal dimension.

In summary, the issue of interpersonal relations, although discussed controversely, is one of the hallmarks of psychiatry and its professional identity. Compulsory interventions, again, present a constant challenge: Without an enduring process of critical reflection, psychiatry will not be in the position to tackle this problem adequately, i.e., to stick to the fine line between overt paternalism and pseudo-liberal negligence.

## Concluding Remarks

Psychiatry is a practical medical field. Therefore, in line with Karl Jaspers’ position in the quotation above, philosophical considerations in psychiatry will only be of value if they create a more profound understanding of diagnostic and therapeutic processes. They cannot reduce the clinician’s burden, but—in the best case—they will make his or her decisions clearer and more substantial.

This leads to four main conclusions:

– Compulsory interventions severely restrict constitutional rights of the patients. Under no circumstances may they be regarded as undisputed “regular” constituents of psychiatric work. They are exceptional measures only to be considered under strict and clearly defined ethical and juridical conditions.
– Compulsory interventions confront mental health professionals with difficult and not seldom provocative questions that challenge their individual professional identity as well as the identity of psychiatry in general. Since there is no “autonomy light,” these debates are demanding endeavors. They have to be integral parts of psychiatry and cannot be fully delegated to external experts or institutions, e.g., ethical councils.
– Guidelines about compulsory interventions and their prevention are highly useful. However, they alone cannot resolve the ethical dilemma brought forward by every single case. Clinicians must stay aware of their indispensable responsibilities.
– The debate about autonomy in psychiatry will serve as an effective and credible point of contact with society, since autonomy also is a central topic in social and political contexts. This common interest may stimulate dialogues and, in the best case, help to fight discrimination of psychiatric patients, professionals, and the field itself.

## Author Contributions

PH is the sole author of this manuscript.

## Conflict of Interest

The author declares that the research was conducted in the absence of any commercial or financial relationships that could be construed as a potential conflict of interest.

## Footnotes

1 In Switzerland, exactly these mandatory prerequisites of any medical intervention that has to be paid for by basic insurance are explicitly mentioned in the federal law on health insurance (“;Krankenversicherungsgesetz,”; KVG). Since this law came into effect in 1994, the “;WZW-criteria”; (for the German terms “;Wirksamkeit, Zweckmaessigkeit, Wirtschaftlichkeit”;) gained considerable influence on health policy and on the conceptualization of health services also in psychiatry and psychotherapy.

2 See footnote 8.

3 This also has a juridical dimension: If a patient—whether capable of decision making or not—is denied necessary medical interventions he or she is entitled to, the psychiatrist might be accused of “;nonassistance of a person in danger.”;

4 Also termed “;power of judgement”; (“;Urteilsfaehigkeit”; in German, “;capacité de discernement”; in French).

5 There are exceptions from this rule: In Switzerland, for example, the lack of the power of judgement is *not* explicitly mentioned as precondition of an involuntary admission in case of a mental disorder (20).

6 Art. 434 Swiss Civil Code (“;Treatment without consent”;)

7 Even more this is true for Johann Gottlieb Fichte (1762–1814) who further developed Kantian principles and defined the interpersonal realm as one of the cornerstones of his philosophical system (21).

8
*To assist* and *not to substitute* autonomy, wherever possible, is a central postulate of the United Nations’ “;Convention on the Rights of Persons with Disabilities”; (3).
